# Андрогенный статус мужчин на фоне COVID-19

**DOI:** 10.14341/probl13077

**Published:** 2022-04-17

**Authors:** Р. В. Роживанов, Г. А. Мельниченко, Е. Н. Андреева, Н. Г. Мокрышева

**Affiliations:** Национальный медицинский исследовательский центр эндокринологии; Национальный медицинский исследовательский центр эндокринологии; Национальный медицинский исследовательский центр эндокринологии; Национальный медицинский исследовательский центр эндокринологии

**Keywords:** тестостерон, гипогонадизм, COVID-19, мужчины

## Abstract

**ОБОСНОВАНИЕ:**

ОБОСНОВАНИЕ. COVID-19 является заболеванием, оказывающим негативное системное воздействие на организм человека, в том числе на мужские гонады. Следовательно, андрогенный статус мужчин при COVID-19 нуждается в изучении.

**ЦЕЛЬ:**

ЦЕЛЬ. Оценить уровни общего тестостерона, глобулина, связывающего половые гормоны (ГСПГ), и свободного тестостерона у мужчин в острой фазе COVID-19 и при реконвалесценции.

**МАТЕРИАЛЫ И МЕТОДЫ:**

МАТЕРИАЛЫ И МЕТОДЫ. Сплошное динамическое проспективное исследование 70 мужчин со среднетяжелой и тяжелой формой COVID-19 в возрасте 50 [44; 64] лет. При проведении исследования определялись уровни общего тестостерона, ГСПГ с дальнейшим расчетом уровня свободного тестостерона по Vermeullen. Данные собирались двукратно — при госпитализации пациента и при его выписке. Статистически значимыми считали различия между группами при p<0,05.

**РЕЗУЛЬТАТЫ:**

РЕЗУЛЬТАТЫ. На момент госпитализации по поводу COVID-19 синдром гипогонадизма отмечался у 61 человека — 87%. Пациенты с гипогонадизмом статистически значимо не различались по возрасту и степени тяжести заболевания COVID-19 по сравнению с мужчинами без гипогонадизма. Стационарное лечение, продолжавшееся 12 [10; 14] дней, привело к статистически значимому увеличению уровней общего тестостерона с 4,7 [2,96; 8,48] до 12,85 [8,62; 19,2] нмоль/л, p<0,001; ГСПГ — с 27,87 [20,78; 36,57] до 33,76 [26,27; 52,60] нмоль/л, p<0,001 и свободного тестостерона с 107 [65; 174] до 235 [162; 337] пмоль/л, p<0,001. Это привело к устранению гипогонадизма у 28 (40%) пациентов. Пациенты с персистенцией гипогонадизма были статистически значимо старше мужчин с нормализацией тестостерона (58 [50; 74] против 45 [40; 52] лет, р<0,001), статистически значимых различий в исходных уровнях общего тестостерона, ГСПГ и свободного тестостерона выявлено не было, также не было различий в распространенности тяжелой формы COVID-19 (3,97 [2,86; 7,46] против 4,26 [2,93; 5,96] нмоль/л, p=0,100; 28,76 [20,78; 48,59] против 24,63 [18,85; 31,70] нмоль/л, р=0,994; 100 [58; 118] против 96 [64; 143] нмоль/л, p=0,522; 24 против 18%, p=0,754, соответственно).

**ЗАКЛЮЧЕНИЕ:**

ЗАКЛЮЧЕНИЕ. COVID-19 оказывает выраженное негативное влияние на выработку тестостерона у мужчин, приводя к развитию лабораторного гипогонадизма, который является потенциально обратимым. Обратимость лабораторного гипогонадизма характерна для более молодых пациентов.

## Abstract (en)

**BACKGROUND:**

BACKGROUND. COVID-19 is a disease that has a negative systemic effect on the human body, including the male gonads. Therefore, the androgenic status in men with COVID-19 needs to be studied.

**AIM:**

AIM. To evaluate the levels of total testosterone, sex hormone binding globulin (SHBG) and free testosterone in men in the acute phase of COVID-19 and during convalescence.

**MATERIALS AND METHODS:**

MATERIALS AND METHODS. A continuous dynamic prospective study of 70 men with moderate to severe COVID-19 at the age of 50[44; 64] years. During the study, the levels of total testosterone, SHBG were determined with further calculation of the level of free testosterone by Vermeullen. The data were collected twice — at the patient’s hospitalization and at his discharge. The differences between the groups were considered statistically significant at p <0.05.

**RESULTS:**

RESULTS. At the time of hospitalization for COVID-19, hypogonadism syndrome was observed in 61 people — 87%. Patients with hypogonadism did not statistically significant differ in age and severity of COVID-19 disease compared to men without hypogonadism. Inpatient treatment lasting 12[10;14] days resulted in a statistically significant increase in the levels of total testosterone from 4,7[2,96;8,48] to 12,85[8,62;19,2] nmol/l, p<0,001; SHBG from 27,87[20,78;36,57] to 33,76[26,27;52,60] nmol/l, p<0,001 and free testosterone from 107[65;174] to 235[162;337] pmol/l, p<0,001. This led to the elimination of hypogonadism in 28 patients — 40%. Patients with persistent hypogonadism were statistically significantly older than men with normalized testosterone, there were no statistically significant differences in the initial levels of total testosterone, SHBG and free testosterone, and there were also no differences in the prevalence of severe COVID-19 (3,97[2,86;7,46] vs 4,26[2,93;5,96] nmol/l, p=0,100; 28,76[20,78;48,59] vs 24,63[18,85;31,70] nmol/l, р=0,994; 100[58;118] vs 96[64;143] pmol/l, p=0,522; 24 vs 18%, p=0,754, respectively).

**CONCLUSION:**

CONCLUSION. COVID-19 has a pronounced negative effect on the production of testosterone in men, leading to the development of laboratoric hypogonadism, which is potentially reversible. The reversibility of laboratoric hypogonadism is typical for younger patients.

## ОБОСНОВАНИЕ

В конце 2019 г. в мире появилась новая коронавирусная инфекция, которая была впервые выявлена в Китае, а ее возбудителю дано название — SARS-CoV-2 [1]. С конца января 2020 г. случаи заболевания регистрировались уже по всему миру, и ВОЗ объявила о наличии пандемии [2][3]. С самого начала развития эпидемии COVID-19 было отмечено, что мужчины заболевают чаще женщин, при этом у них отмечаются более тяжелые формы проявления инфекции [4]. Точные причины такой ситуации не установлены, но, возможно, это связано с наличием каких-то особенностей, делающих мужчин более восприимчивыми к коронавирусу, например, андрогенный статус [4]. Так, было отмечено, что те пациенты, которые получают андрогендепривационную терапию, реже заболевают и легче переносят SARS-CoV-2, и этот эффект исследователи объясняют воздействием на белок TMPRSS2, синтез которого является андрогензависимым [5]. В других исследованиях было установлено, что как экспрессия TMPRSS2, так и более тяжелое течение коронавирусной инфекции отмечаются у мужчин с гиперандрогенией — андрогенной алопецией, акне, выраженным лицевым оволосением и повышенной жирностью кожи [6–8]. Однако в исследовании G. Rastrelli и соавт. (2020) было показано, что низкие уровни тестостерона связаны с худшим прогнозом коронавирусной инфекции у мужчин [9]. Такой же вывод был сделан в результате недавнего отечественного исследования [10]. В другой работе отмечено, что наличие в анамнезе COVID-19 увеличивает риск развития гипогонадизма [11]. Все это обуславливает необходимость изучить динамику выработки тестостерона на фоне COVID-19.

## ЦЕЛЬ ИССЛЕДОВАНИЯ

Оценить уровни общего тестостерона, глобулина, связывающего половые гормоны (ГСПГ), и свободного тестостерона у мужчин в острой фазе COVID-19 и при реконвалесценции.

## МАТЕРИАЛЫ И МЕТОДЫ

## Место и время проведения исследования

ФГБУ «НМИЦ эндокринологии» Министерства здравоохранения РФ, Москва. Исследование выполнено в период с мая 2020 по август 2021 г.

## Изучаемые популяции

Формирование групп проводилось из пациентов, госпитализированных в ФГБУ «НМИЦ эндокринологии» Министерства здравоохранения РФ в связи с COVID-19.

Критериями включения являлись мужской пол, возраст старше 18 лет, тяжелая и среднетяжелая формы COVID-19.

Критерия невключения не предусматривались.

Критерии исключения — отказ пациента от участия в исследовании, смерть пациента в исходе COVID-19 и, следовательно, невозможность оценить динамику изучаемых параметров.

Всего в исследование были включены 70 пациентов, возраст 50 [ 44; 64] лет.

## Способ формирования выборки из изучаемой популяции

Выборка формировалась сплошным способом.

## Дизайн исследования

Пилотное одноцентровое наблюдательное динамическое проспективное одновыборочное неконтролируемое несравнительное исследование.

## Методы

При проведении исследования проводились диагностика и лечение COVID-19 в соответствии с клиническими рекомендациями [1]. Определялись уровни общего тестостерона (норма 12,0–30,0 нмоль/л) и ГСПГ (норма 12–65 нмоль/л) на автоматическом анализаторе Vitros ECi (Johnson and Johnson, Великобритания) методом усиленной хемилюминесценции. Уровень свободного тестостерона определялся расчетным методом по Vermeullen (норма 243–900 пмоль/л) [12]. В работе не оценивались клинические симптомы гипогонадизма, выявлялся лабораторный гипогонадизм, интерпретация референса основывалась на клинических рекомендациях [13][14]. Забор крови для исследования исходно проводился на 9 [ 7; 12] день от начала первых симптомов заболевания, а в динамике — на 21 [ 18; 26] день от начала первых симптомов заболевания.

## Статистический анализ

Принципы расчета размера выборки

Исследование пилотное и сплошное. Размер выборки предварительно не рассчитывался.

Методы статистического анализа данных

Полученные данные обработаны с использованием пакета статистических программ STATISTICA 13.0 (StatSoft Inc., США) [15]. Оценка статистических различий между группами осуществлялась с использованием теста Вилкоксона и U-критерия Манна–Уитни для количественных признаков и точного критерия Фишера для качественных. Различия между группами считались статистически значимыми при р<0,05.

## Этическая экспертиза

Проведение исследования одобрено локальным Этическим комитетом ФГБУ «НМИЦ эндокринологии» Минздрава России (протокол № 6 от 30.04.2021).

## РЕЗУЛЬТАТЫ

Из 70 мужчин, включенных в исследование на момент госпитализации с COVID-19, лабораторный гипогонадизм отмечался у 61 человека — 87%. Пациенты с нормальным уровнем тестостерона (n=9) не имели статистически значимых различий по сравнению с мужчинами с лабораторным гипогонадизмом в возрасте (p=0,456; U-тест Манна–Уитни) и степени тяжести заболевания COVID-19 (p=1,0, точный критерий Фишера). Возраст пациентов с лабораторным гипогонадизмом составил 48 [ 45; 59] лет, а без такового — 51 [ 44; 65] год, распространенность тяжелой формы COVID-19 составила 22 и 21% соответственно. Стационарное лечение COVID-19, продолжавшееся 12 [ 10; 14] дней, привело к статистически значимому увеличению уровней общего тестостерона, ГСПГ и свободного тестостерона (табл. 1).

**Table:** Таблица 1. Содержание тестостерона и ГСПГ в периферической крови у пациентов с лабораторным гипогонадизмом на фоне COVID-19 в острой фазе и после выздоровленияTable 1. Testosterone and SHBG levels in peripheral blood in patients with laboratory hypogonadism on the background of COVID-19 in the acute phase and after recovery Примечания: метод Вилкоксона; данные представлены в виде медиан и границ интерквартильного отрезка (25–75%).

Параметр	Острая фаза (n=70)	Реконвалесценция (n=70)	p
ГСПГ, нмоль/л	27,87 [ 20,78; 36,57]	33,76 [ 26,27; 52,60]	<0,001
Общий тестостерон, нмоль/л	4,7 [ 2,96; 8,48]	12,85 [ 8,62; 19,2]	<0,001
Свободный тестостерон, пмоль/л	107 [ 65; 174]	235 [ 162; 337]	<0,001

Это привело к устранению лабораторного гипогонадизма у 28 пациентов — 40%, таким образом, его распространенность к моменту выписки пациентов из стационара составила 47%. Результаты обследования пациентов в зависимости от персистенции лабораторного гипогонадизма представлены в таблице 2.

**Table:** Таблица 2. Показатели возраста, содержания тестостерона и ГСПГ в периферической крови, распространенности тяжелой формы COVID-19 в зависимости от персистенции лабораторного гипогонадизмаTable 2. Indicators of age, testosterone and SHBG levels in peripheral blood, prevalence of severe COVID-19 depending on the persistence of laboratory hypogonadism Примечания: U-тест Манна–Уитни для количественных признаков, точный критерий Фишера для качественных; данные представлены в виде медиан, границ интерквартильного отрезка (25–75%), процентов.

Параметр	Гипогонадизм устранен (n=28)	Гипогонадизм персистирует (n=33)	p
Возраст, лет	45 [ 40; 52]	58 [ 50; 74]	<0,001
ГСПГ (исходно), нмоль/л	24,63 [ 18,85; 31,70]	28,76 [ 20,78; 48,59]	0,994
Общий тестостерон (исходно), нмоль/л	4,26 [ 2,93; 5,96]	3,97 [ 2,86; 7,46]	0,100
Свободный тестостерон (исходно), пмоль/л	96 [ 64; 143]	100 [ 58; 118]	0,522
Распространенность тяжелой формы COVID-19, %	18	24	0,754

Пациенты с персистенцией лабораторного гипогонадизма и мужчины с достигнутым нормогонадным статусом статистически значимо отличались по возрасту (пациенты с персистенцией гипогонадизма были старше (р<0,001)). Статистически значимых различий в исходных уровнях общего тестостерона, ГСПГ и свободного тестостерона выявлено не было, также не было различий в распространенности тяжелой формы COVID-19 (р=0,100; p=0,994; p=0,522; p=0,754 соответственно).

## ОБСУЖДЕНИЕ

## Репрезентативность выборок

Оценить репрезентативность выборки по отношению к общей популяции не представляется возможным, поскольку формирование выборки проводилось сплошным методом из пациентов, госпитализированных только в один федеральный научный центр.

## Сопоставление с другими публикациями

Распространенность лабораторного гипогонадизма, выявленная в нашем исследовании, является очень высокой и не может быть объяснена только лишь возрастом пациентов, так как распространенность гипогонадизма у мужчин без сопутствующих хронических заболеваний составляет около 5% [16]. Очевидно воздействие переносимого заболевания. И это воздействие на выработку тестостерона является клинически значимым, так как даже при таком заболевании, как сахарный диабет (СД) 2 типа, возникающем в пожилом возрасте, с доказанным негативным влиянием на выработку тестостерона распространенность гипогонадизма составляет от 15 до 50% [17–19]. В Российском эпидемиологическом исследовании, включившем 554 мужчины с СД 2 типа в возрасте 55 [ 50; 58] лет, распространенность гипогонадизма составила 32,7% [20]. Лишь одно из немногих ранее проведенных исследований в России продемонстрировало более высокую распространенность гипогонадизма — 68–83% в зависимости от метода выявления, однако это исследование было ограничено выборкой больных стационара, для которых характерны более тяжелое течение СД и наличие множественных сопутствующих заболеваний. Средний возраст 82 обследованных пациентов составил 53,8 (95% ДИ 52,0–55,7) лет. Распространенность гипогонадизма по данным измерения уровня общего тестостерона крови составила 68,3% и увеличивалась с возрастом: в возрастной группе 40–50 лет — 60%, в возрастной группе 51–60 лет — 71% и в возрастной группе от 61 до 70 лет — 76% [21]. Таким образом, COVID-19 оказывает более выраженное негативное влияние на выработку тестостерона у мужчин даже по сравнению с декомпенсацией СД у пожилых пациентов.

Еще одним заболеванием легочной ткани, негативно влияющим на выработку тестостерона, является хроническая обструктивная болезнь легких (ХОБЛ). В исследовании, включившем 197 мужчин с ХОБЛ, пациенты были разделены на группы с хроническим необструктивным бронхитом, легким, умеренным и тяжелым течением ХОБЛ, компенсированным и декомпенсированным легочным сердцем. В последних четырех группах отмечено снижение уровня тестостерона по сравнению с контролем, при этом распространенность гипогонадизма составила от 22 до 69% [22]. По сравнению с представленными величинами влияние COVID-19 на выработку тестостерона более негативное.

В других работах также показано, что наличие в анамнезе COVID-19 увеличивает риск развития гипогонадизма, а тяжесть течения COVID-19 ассоциирована с увеличением его распространенности [11][23]. Так, в одном из исследований, включившем 143 пациента, выздоровевших от COVID-19, распространенность гипогонадизма составила 28,7%. Обследование проводилось после 77 дней (72–83) от начала болезни, у 72% пациентов отмечалась пневмония [11]. В нашем исследовании пневмония отмечалась у всех пациентов, при этом обследование проводилось не в момент выздоровления, а в острой фазе, что и обусловило большую распространенность гипогонадизма — 87%. К моменту улучшения состояния (выписка из стационара) распространенность гипогонадизма снизилась до 47%.

В другом исследовании пациенты были разделены на две группы: 1-я группа — 35 мужчин со среднетяжелой и тяжелой формой COVID-19 с наличием коморбидностей в возрасте 53,5±14 лет и 2-яvгруппа — 49 мужчин со среднетяжелой формой COVID-19 без коморбидностей в возрасте 31,9±13 лет [23]. Распространенность гипогонадизма в 1-й группе составила 75,6%, что сопоставимо с полученными нами результатами, а во 2-й — 20,6%, что логично, так как течение основного заболевания было более легким, а пациенты моложе.

Кроме того, вызывают интерес результаты отечественного исследования ОСНОВАТЕЛЬ, в котором авторы обследовали 152 мужчин с подтвержденным диагнозом COVID-19 по результатам положительного ПЦР-теста и/или данным компьютерной томографии легких, госпитализированных в связи со среднетяжелым и тяжелым течением инфекции. Было установлено, что среди мужчин с новой коронавирусной инфекцией среднего и тяжелого течения снижение уровня тестостерона выявляется в46,7% случаев, при этом лечение COVID-19 приводило к значимому повышению уровня тестостерона. А отсутствие повышения тестостерона в процессе лечения было ассоциировано с сохранением большего объема поражения легких [10].

## Клиническая значимость результатов

Полученные результаты имеют значение для клинической практики, так как демонстрируют необходимость оценки уровня тестостерона у мужчин с COVID-19 с целью выявления гипогонадизма и дальнейшего мониторинга, а при необходимости — проведения андрогенной терапии.

## Ограничения исследования

Ограничениями исследования являются проблемы с репрезентативностью выборки в отношении общей популяции (сформирована только из пациентов крупного федерального центра), а также использование в исследовании непрямого метода определения свободного тестостерона, а расчетной методики. Поскольку в работе не проводилось вирусологического исследования тестикулярной ткани, сложно заявлять, являются ли наблюдаемые эффекты следствием поражения коронавирусом клеток Лейдига или обусловлены инфекционно-воспалительным процессом как таковым, однако обратимость лабораторного гипогонадизма позволяет предположить, скорее, воздействие общего инфекционно-воспалительного процесса.

## Направления дальнейших исследований

В продолжение проведенного исследования планируется изучить эффективность и безопасность андрогенной заместительной терапии у мужчин с персистенцией гипогонадизма после COVID-19.

## ЗАКЛЮЧЕНИЕ

COVID-19 оказывает выраженное негативное влияние на выработку тестостерона у мужчин, приводя к развитию лабораторного гипогонадизма, который является потенциально обратимым. Обратимость лабораторного гипогонадизма характерна для более молодых пациентов. Пациенты с персистенцией гипогонадизма могут являться кандидатами для проведения андрогенной терапии.

## ДОПОЛНИТЕЛЬНАЯ ИНФОРМАЦИЯ

Источник финансирования. Исследование осуществлено на спонсорские средства фирмы ООО «Безен Хелскеа РУС» (123022, Москва, ул. Сергея Макеева, 13).

Конфликт интересов. Роживанов Р.В. — чтение образовательных лекций для фирмы ООО «Безен Хелскеа РУС» (123022, Москва, ул. Сергея Макеева, 13); Мельниченко Г.А., Андреева Е.Н., Мокрышева Н.Г. подтвердили отсутствие конфликта интересов, о котором необходимо сообщить.

Выражение признательности. Авторы выражают искреннюю благодарность пациентам, принявшим участие в проведении исследования.

Участие авторов. Роживанов Р.В. — существенный вклад в получение, анализ данных и интерпретацию результатов, написание статьи; Мельниченко Г.А. — существенный вклад в концепцию и дизайн исследования, интерпретацию результатов; Андреева Е.Н. — существенный вклад во внесение правки в рукопись с целью повышения научной ценности статьи; Мокрышева Н.Г. — существенный вклад во внесение правки в рукопись с целью повышения научной ценности статьи.
